# Differential effect of NMDA receptor GluN2C and GluN2D subunit ablation on behavior and channel blocker-induced schizophrenia phenotypes

**DOI:** 10.1038/s41598-019-43957-2

**Published:** 2019-05-20

**Authors:** Gajanan P. Shelkar, Ratnamala Pavuluri, Pauravi J. Gandhi, Aparna Ravikrishnan, Dinesh Y. Gawande, Jinxu Liu, Dustin J. Stairs, Rajesh R. Ugale, Shashank M. Dravid

**Affiliations:** 1 Department of Pharmacology, Creighton University, Omaha, NE 68178 USA; 2 Psychology, Creighton University, Omaha, NE 68178 USA; 3 Department of Pharmaceutical Sciences, R.T.M. Nagpur University, Nagpur, Maharashtra 440033 India

**Keywords:** Ion channels in the nervous system, Sensorimotor processing

## Abstract

The GluN2C- and GluN2D-containing NMDA receptors are distinct from GluN2A- and GluN2B-containing receptors in many aspects including lower sensitivity to Mg^2+^ block and lack of desensitization. Recent studies have highlighted the unique contribution of GluN2C and GluN2D subunits in various aspects of neuronal and circuit function and behavior, however a direct comparison of the effect of ablation of these subunits in mice on pure background strain has not been conducted. Using knockout-first strains for the *GRIN2C* and *GRIN2D* produced on pure C57BL/6N strain, we compared the effect of partial or complete ablation of GluN2C and GluN2D subunit on various behaviors relevant to mental disorders. A large number of behaviors described previously in GluN2C and GluN2D knockout mice were reproduced in these mice, however, some specific differences were also observed possibly representing strain effects. We also examined the response to NMDA receptor channel blockers in these mouse strains and surprisingly found that unlike previous reports GluN2D knockout mice were not resistant to phencyclidine-induced hyperlocomotion. Interestingly, the GluN2C knockout mice showed reduced sensitivity to phencyclidine-induced hyperlocomotion. We also found that NMDA receptor channel blocker produced a deficit in prepulse inhibition which was prevented by a GluN2C/2D potentiator in wildtype and GluN2C heterozygous mice but not in GluN2C knockout mice. Together these results demonstrate a unique role of GluN2C subunit in schizophrenia-like behaviors.

## Introduction

N-methyl-D-aspartate receptors (NMDARs) are inotropic glutamate receptors that mediate excitatory neurotransmission in the central nervous system. The NMDARs are heterotetrameric complexes composed of two obligatory GluN1 subunits and generally two GluN2 subunits of which there are four types GluN2A–GluN2D. The various GluN2 subunits have unique developmental and cell-type specific expression patterns^1–8^. Subunit composition determines the electrophysiological and pharmacological properties of NMDARs. The GluN2C- and GluN2D-containing receptors have lower sensitivity to Mg^2+^-block compared to GluN2A- and GluN2B-containing receptors, lack desensitization and have high affinity for glutamate and glycine which may allow their activation by spillover glutamate^1,9,10^. Although similar in several aspects, GluN2C- and GluN2D-containing receptors also diverge in several biophysical and pharmacological properties. For example, in the presence of Mg^2+^, GluN1/GluN2C receptors exhibit higher blockade with ketamine compared to GluN1/GluN2D receptors or other NMDAR subtypes^11^. This differential channel blocker affinity has been proposed to contribute to the psychotic symptoms in humans^11–13^. Additionally, ketamine produces antidepressant effect in patients with treatment resistant-depression and shows efficacy in patients with refractory epilepsy^14–16^ but its subunit-selectivity in these actions, if any, remains poorly understood.

There were two goals of this study. First to conduct a side-by-side comparison of GluN2C and GluN2D knockout (KO) mice models in the same sub-strain of mice. We and others have previously conducted behavioral characterization of GluN2C KO mice^17–19^ and GluN2D KO mice^20–25^. However, the mouse strains among these studies were variable. In the present study, we obtained GluN2C and GluN2D knockout-first mice lines which were both on pure C57BL/6N background and were generated using similar strategy by insertion of a reporter cassette in the *GRIN2C* and *GRIN2D* genes. We compared the behavioral effect of complete or partial ablation of GluN2C or GluN2D. The second goal of the study was to address the role of GluN2C and GluN2D in channel blocker effects using KO models.

We found that GluN2D KO mice showed hypolocomotion as well as anxiety-like behavior. We also observed specific increase in startle amplitude in GluN2C heterozygous (GluN2C HET) and GluN2D HET and KO animals. GluN2C and GluN2D KO mice showed depression-like behavior in forced swim test. Importantly, unlike previous studies^22–24^ we found that GluN2D KO mice were not resistant to NMDA receptor channel blocker-induced hyperlocomotion. In contrast, GluN2C KO mice exhibited reduced sensitivity to NMDAR channel blocker-induced hyperlocomotion. Recent studies have identified subunit-selective modulators for GluN2C/2D-containing receptors. CIQ is a positive allosteric modulator of GluN2C/2D-containing NMDA receptors and increases frequency of channel opening but not channel mean open time^26^. CIQ does not potentiate currents through GluN2A/2B-containing NMDA receptors. CIQ binding site has been identified to the transmembrane domain and the pre-M1 helix region of the GluN2 subunit^27^. The selectivity of CIQ for GluN2C/2D-containing receptors may arise due to the transfer of conformational changes by the linker between the ligand binding domain and amino terminal domain^26^. In addition to the NMDA receptors, CIQ also exhibits binding to the nicotinic receptors^28^ but the functional effect of this binding is not known. We have previously shown that CIQ a positive allosteric modulator of GluN2C/2D receptors reverses NMDA channel blocker-induced deficit in prepulse inhibition^29^ which is a model of sensorimotor deficits in schizophrenia. Here we further found that CIQ prevented MK-801-induced PPI deficit in wildtype and GluN2C HET mice but not in GluN2C KO suggesting a requirement of GluN2C subunit in the efficacy of CIQ. Together, these studies indicate a critical role of GluN2C in schizophrenia-like behaviors.

## Results

### GluN2D knockout mice exhibit hypolocomotion and anxiety-like behavior

**Figure 1:**
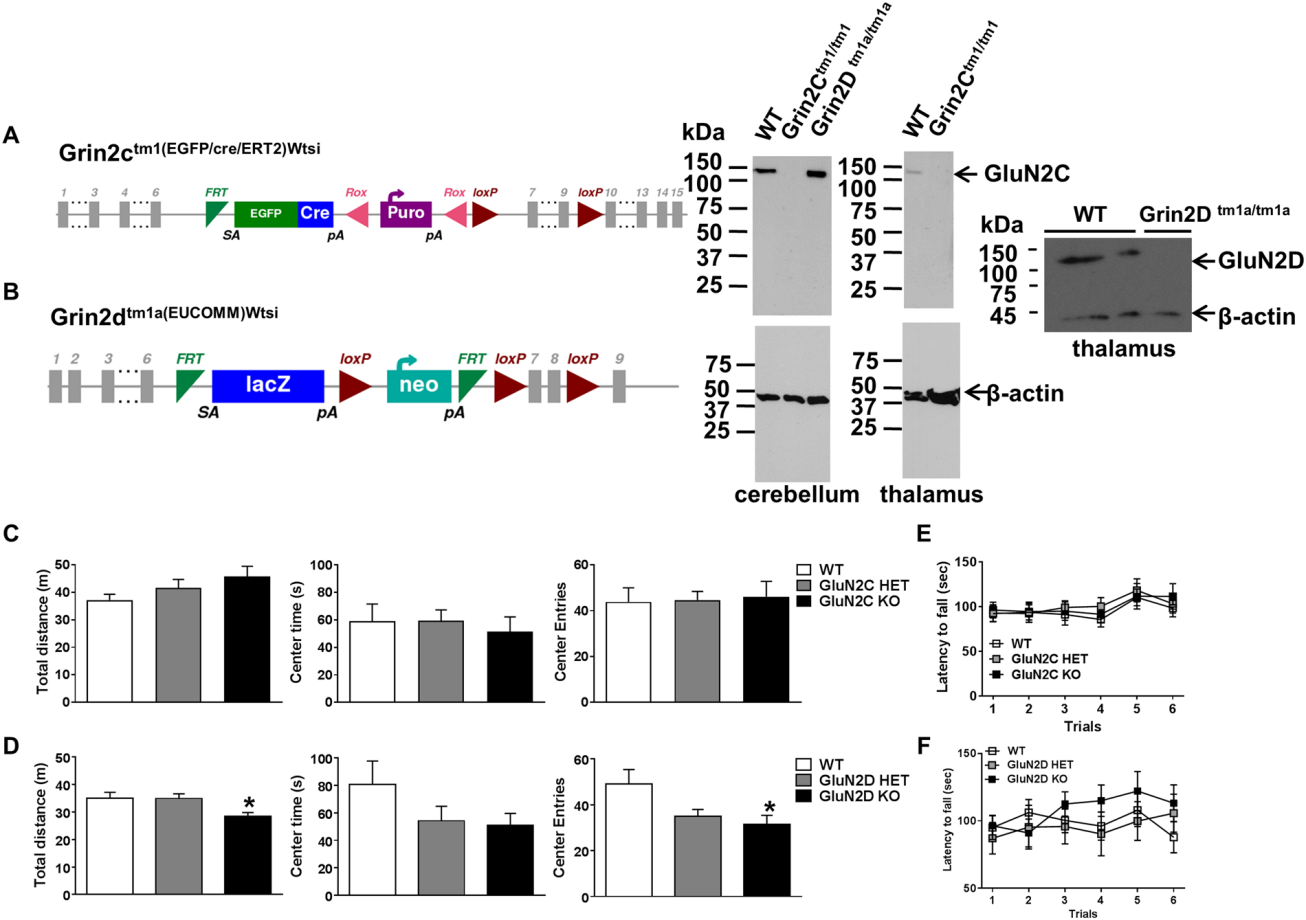
GluN2D knockout mice exhibit hypolocomotion and anxiety-like behavior. (**A**) Diagram showing the gene construct for GluN2C KO mice (MGI Ref: J:155845, left panel). Western blot showing the expression of GluN2C in synaptosomal protein from cerebellum and thalamus (right panel). Lack of immunoreactivity in GluN2C KO but not in WT and GluN2D KO confirming the specific deletion of GluN2C in KO animals. (**B**) Showing the gene construct for GluN2D KO mice (MGI Ref: J:155845, J:173534, left panel). Western blot showing the expression of GluN2D in synaptoneurosome from thalamus (right panel). Lack of immunoreactivity in GluN2D KO animals confirms the deletion of GluN2D subunits. The images represent the entire unmodified (for exposure or contrast) blots of the indicated samples from ~250 to ~40 kDa. (**C**) WT (n = 12), GluN2C HET (n = 13) and KO (n = 7) mice were tested in open field. Partial or complete ablation of GluN2C subunits did not affect the total distance traveled, time spent in central square or number of entries into the center square. (**D**) Open field analysis for WT (n = 13), GluN2D HET (n = 22) and KO (n = 22) mice. GluN2D KO mice exhibited lower total distance traveled as well as lower number of entries into the center square. (**E**) WT (n = 8), GluN2C HET (n = 11) and KO (n = 13) mice were tested for motor coordination and learning in rotarod test. No significant deficit in motor coordination and learning was observed following ablation of GluN2C subunits. (**F**) No significant difference was noted in the WT (n = 12), GluN2D HET (n = 7) and KO (n = 14) mice in the rotarod test. Data represented as mean ± SEM. *P < 0.05, one-way ANOVA followed by Bonferroni post-hoc test.

We validated the two knockout-first models by conducting western blot analysis for protein expression. We collected cerebellar and thalamic synaptosomal protein and tested the expression of GluN2C subunit using the Neuromab antibody. GluN2C band was observed at the appropriate size (~130 kDa) in wildtype and GluN2D KO but not in GluN2C KO confirming that the knockout-first indeed lacked GluN2C expression (Fig. 1A). We further tested thalamic synaptoneurosome samples from wildtype and GluN2D KO mice using a GluN2D antibody from Millipore. A specific band was observed at ~140 kDa in wildtype but not in GluN2D KO confirming the knockout-first model lacked GluN2D expression (Fig. 1B). The β-actin band was observed in all samples.

Open field test was conducted in HET and KO mice for GluN2C and GluN2D subunit. Total distance traveled, and the number of entries and time spent in the center zone of the open field arena, a measure of anxiety-like behavior, was scored. No significant difference in total distance traveled or center time or entries was observed in GluN2C HET or GluN2C KO mice (One-way ANOVA). In contrast, GluN2D KO mice showed significantly lower distance traveled [F (2,55) = 5.549, P = 0.006 one-way ANOVA followed by Bonferroni’s test, Fig. 1D] and number of entries into the central zone [F (2,55) = 4.127, P = 0.02, one-way ANOVA followed by Bonferroni’s test, Fig. 1D]. A trend for reduced time spent in the central square was observed in GluN2D KO mice and trend for reduced time spent and entries in central square was also observed in GluN2D HET. Thus, ablation of GluN2D subunit reduces exploratory locomotor activity and partial or complete ablation of GluN2D subunit potentially leads to higher anxiety-like behavior. We further analyzed the effect of GluN2C and GluN2D ablation on motor coordination and learning using the rotarod test. No significant differences amongst the wildtype, HET and KO animals in either GluN2C (Fig. 1E) or GluN2D (Fig. 1F) genotype was observed in rotarod test (repeated measures ANOVA).

### GluN2C and GluN2D deletion affects startle response

**Figure 2:**
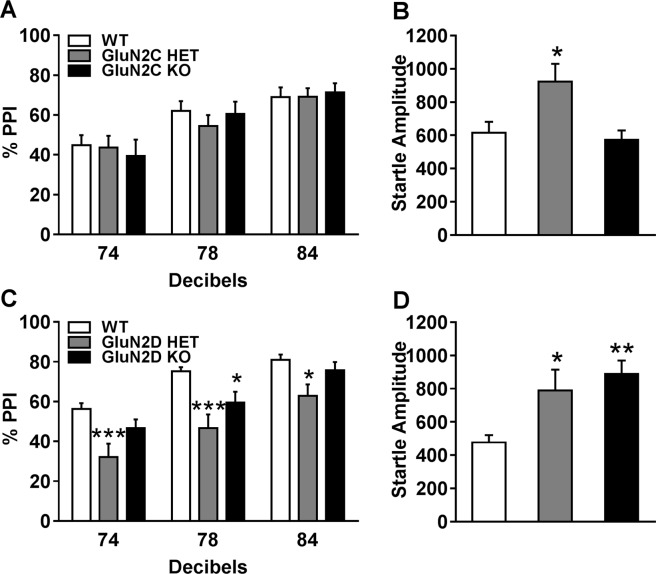
Effect of GluN2C and GluN2D deletion on prepulse inhibition and startle response. (**A**) PPI were recorded in WT (n = 17), GluN2C HET (n = 15) and KO (n = 10). No change in PPI was noted in the GluN2C HET or KO. (**B**) An increase in startle amplitude was found in GluN2C HET mice (P = 0.0261, one-way ANOVA followed by Bonferonni post-hoc test). (**C**) PPI in the WT (n = 19), GluN2D HET (n = 11) and KO (n = 15) mice. Significant reduction in PPI was found in GluN2D HET (74 dB, P = 0.0006, 78 dB, P < 0.0006, and 84 dB, P = 0.0142) and KO (78 dB, P = 0.022, two-way repeated measures ANOVA followed by Bonferonni post-hoc test). *P < 0.05, ***P < 0.001 compared to WT. (**D**) An increase in startle amplitude was found in GluN2D HET (P = 0.02) and KO P = 0.0007, One-way ANOVA followed by Bonferonni post-hoc test) as compared to WT mice. *P < 0.05, ***P < 0.001 compared to WT.

Prepulse inhibition (PPI) of the startle response is a measure of sensorimotor gating, which is impaired in certain psychiatric disorders, and specifically in schizophrenia^30–32^. We have previously found a significant increase in startle response in GluN2C HET and KO mice which were on a mixed background. Similar trend for an increase has been previously reported^33^. Furthermore, GluN2D KO mice also have higher startle response^24,33^. In contrast, ablation of GluN2C and GluN2D subunits has a relatively small effect on PPI. We assessed the effect of complete or partial deletion of GluN2C or GluN2D subunits on PPI in mice on pure C57BL/6N background. No significant differences were observed in the % PPI in GluN2C HET and KO animals (Fig. 2A) but a significant increase in the startle amplitude was found in GluN2C HET [F (2,39) = 5.146, P = 0.02, one-way ANOVA followed by Bonferroni post-hoc test, Fig. 2B] but not GluN2C KO mice. Significant differences were observed in % PPI in GluN2D HET and KO compared to WT animals (Fig. 2C). Two-way repeated measures ANOVA revealed a significant effect of decibel [F (2, 84) = 111, P < 0.0001)] and genotype [F (2, 42) = 8.673, P = 0.0007)] on % PPI response (Fig. 2C). Bonferroni’s post-hoc test revealed a significant reduction in PPI response in the HET (74 dB; P = 0.0006, 78 dB; P < 0.0001, 84 dB; P = 0.014) and KO (78 dB; P = 0.022). One-way ANOVA showed significant effect of GluN2D genotype on the startle amplitude [F(2,42) = 8.845, P = 0.0006, Fig. 2D]. Post-hoc Bonferroni’s test showed significant increase in the startle amplitude in both GluN2D HET (P = 0.0249) and GluN2D KO (P = 0.0007) (Fig. 2D).

### Deletion of GluN2C and GluN2D leads to depression-like behavior

**Figure 3:**
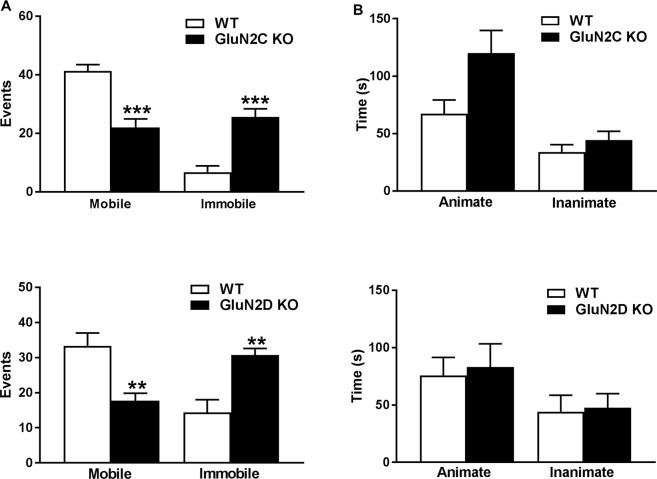
GluN2C and GluN2D KO mice exhibit depression-like behavior but no social deficit. (**A**) Depression-like behavior was assessed using forced swim test in WT (n = 7) and GluN2C KO (n = 9) and WT (n = 9) and GluN2D KO (n = 7) mice. The KO from both the genotypes showed significant reduction in the mobile (P = 0.0001, P = 0.004 for GluN2C and GluN2D respectively, Unpaired t-test) and an increase in immobile events (P = 0.0001 and P = 0.002, for GluN2C and GluN2D respectively, Unpaired t-test) as compared to WT mice for respective parameter. **P < 0.01, **P < 0.01, ***P < 0.001. (**B**) No social deficit was observed in GluN2C KO (n = 5) compared to WT (n = 7). Similarly, in GluN2D KO (n = 8) mice we did not find any significant difference in sociability test as compared to WT mice (n = 8).

We next tested depression-like behavior in GluN2C and GluN2D KO mice using the forced swim test. GluN2C KO mice were found to exhibit significantly lower mobile (P = 0.0001) and higher immobile events (P = 0.0001, Unpaired t-test, Fig. 3A) as compared to wildtype animals. Similarly, GluN2D KO mice showed significantly lower mobile (P = 0.004) and higher immobile events (P = 0.002) as compared to wildtype animals (Unpaired t-test). We next tested anti-depressant efficacy of ketamine in the GluN2C and GluN2D KO mice. We did not find significant effect of ketamine in wildtype mice (data not shown) and therefore genotype effects could not be compared. Next, we examined whether deletion of GluN2C or GluN2D leads to changes in social behavior. We performed sociability test as previously described^34^. Although no significant effect of ablation of GluN2C and GluN2D subunits was observed in social behavior, a trend for an increase in time spent near the animate object was found in the GluN2C and GluN2D KO mice (Fig. 3B).

### GluN2C KO mice have reduced susceptibility to phencyclidine-induced hyperlocomotion

**Figure 4:**
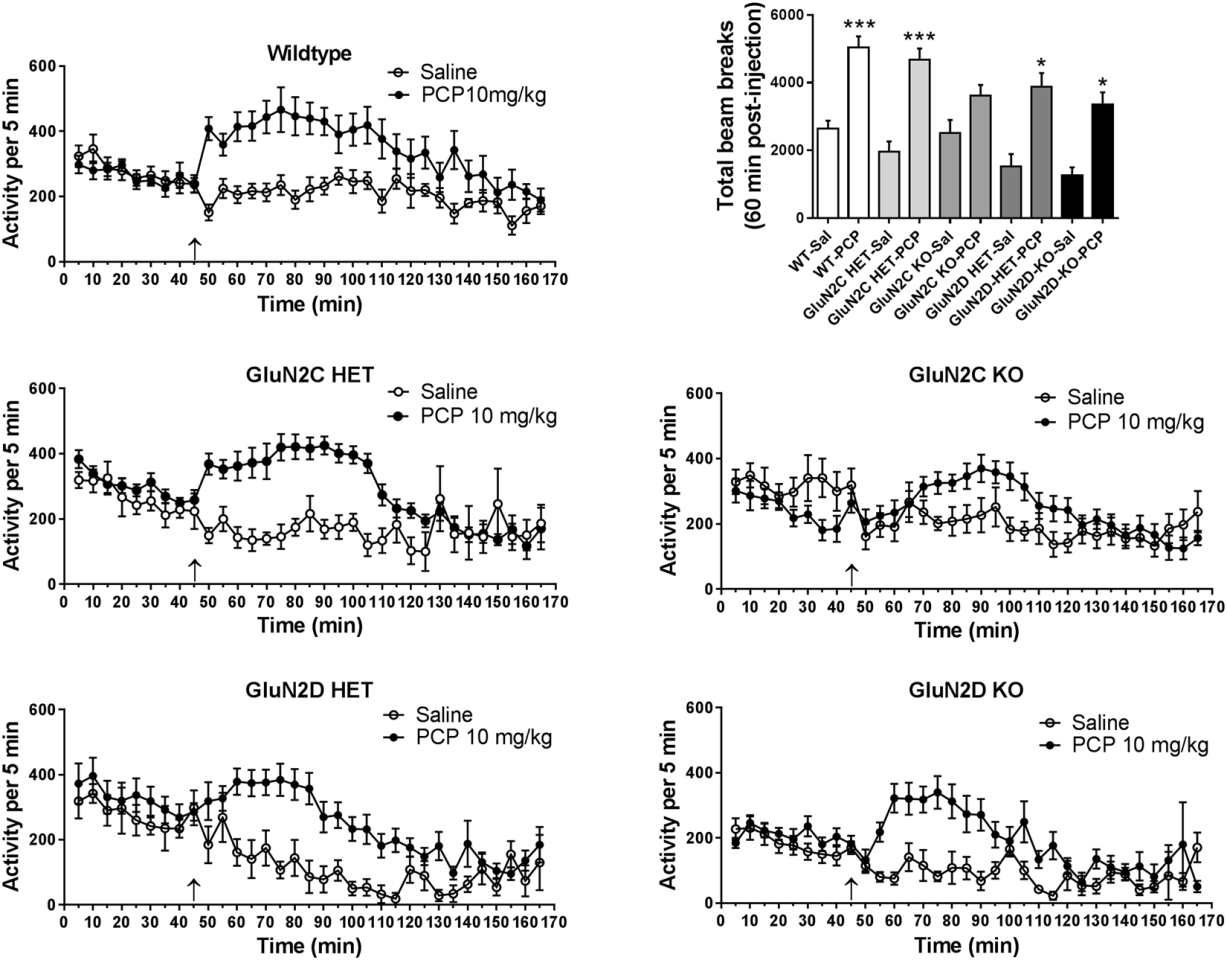
Effect of NMDA channel blocker phencyclidine (PCP) on locomotor activity. Wildtype (Saline: n = 12, PCP: n = 14), GluN2C HET (Saline: n = 8, PCP: n = 19), KO (Saline: n = 9, PCP: n = 12) and GluN2D HET (Saline: n = 5, PCP: n = 20) and KO (Saline: n = 8, PCP: n = 9) mice were treated with saline or PCP (10 mg/kg, ip) and assessed for locomotor activity. Time course of pre- and post-PCP administration locomotor activity for each genotype is depicted. Total locomotor activity for 60 minutes post-PCP injection was calculated and compared. Wildtype (P = 0.0002), GluN2C HET (P < 0.0001), GluN2D HET (P = 0.0156) and GluN2D KO (P = 0.343) but not GluN2C KO showed significant increase in locomotor activity following PCP treatment. *P < 0.05, ***P < 0.001 vs respective saline control, one-way ANOVA followed by Bonferonni post-hoc test.

We further tested the effect of NMDA receptor channel blocker phencyclidine (PCP) on locomotor activity. After a drug-free period of 45 minutes, wildtype, GluN2C or GluN2D HET or KO mice received an injection of saline or PCP (10 mg/kg, intra-peritoneal (ip)) and thereafter the locomotor activity was scored for 120 min. One-way ANOVA showed significant effect of PCP treatment on locomotor activity (F (9,107) = 10.92, P < 0.0001, Fig. 4). Application of post-hoc Bonferroni’s multiple comparison test showed that the PCP significantly increased locomotor activity in wildtype (P = 0.0002) and GluN2C HET (P < 0.0001) as compared to respective saline treatment. Similarly, GluN2D HET (P = 0.0156) and GluN2D KO showed significant hyperlocomotion (P = 0.0343) following PCP treatment as compared to respective saline treated animals. Among all the groups it appeared that GluN2C KO mice were least susceptible to PCP-induced hyperlocomotion since PCP did not produce a significant change in locomotor activity in GluN2C KO as compared to saline controls (P > 0.99 GluN2C KO saline versus PCP, one-way ANOVA).

**Figure 5:**
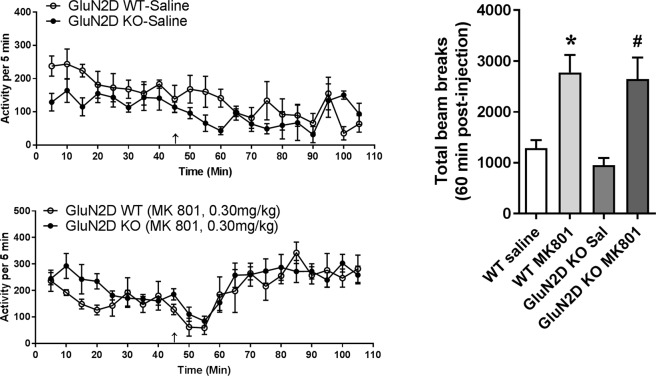
GluN2D KO mice are not resistant to MK-801-induced hyperlocomotion. WT (Saline: n = 5, MK-801: n = 11) and GluN2D KO (Saline: n = 4, MK-801: n = 5) mice were treated with saline or MK-801 (0.3 mg/kg, ip) and tested for locomotor activity. MK-801 significantly increased the locomotor activity in WT (*P = 0.01 compared to the WT saline, Unpaired t-test). GluN2D KO showed a similar increase in locomotor activity compared to saline treated GluN2D KO mice (^#^P = 0.01 compared to the WT saline, Unpaired t-test).

In order to confirm that lack of resistance of GluN2D KO mice to PCP was not dependent on the specific channel blocker, we tested the effect of another NMDA channel blocker MK-801 (0.3 mg/kg, ip) in wildtype and GluN2D KO mice. Similar to the PCP, treatment with MK-801 produced significant increase in the locomotor activity in wildtype mice (P = 0.01, Unpaired t-test, Fig. 5). Importantly, a similar level of hyperlocomotion was observed in GluN2D KO mice (P = 0.007, Unpaired t-test) compared to saline control and the time-dependent locomotor activity after MK-801 was similar in wildtype and GluN2D KO mice.

### GluN2C subunit is necessary for rescue of PPI deficit by GluN2C/2D potentiator CIQ

**Figure 6:**
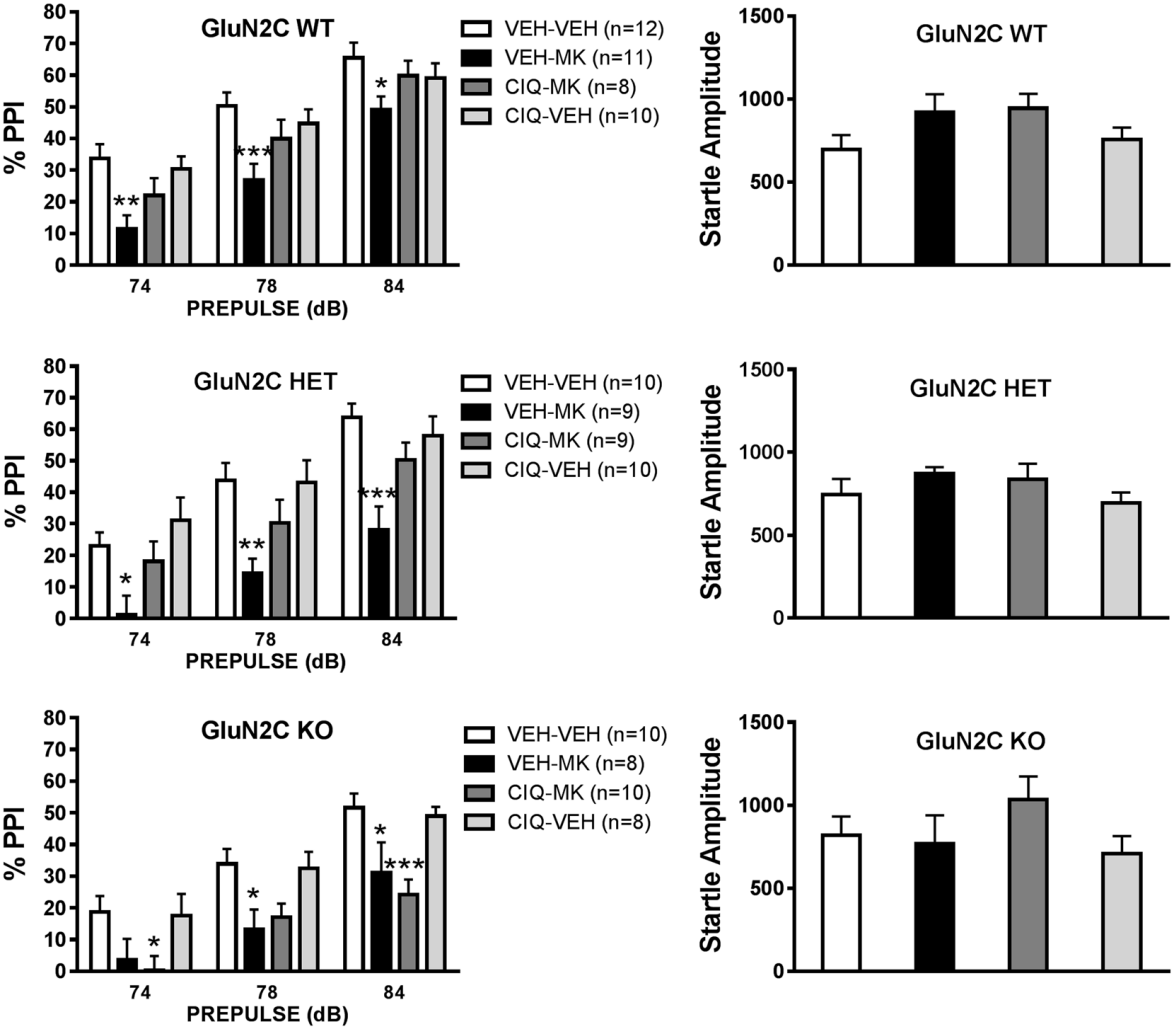
Effect of GluN2C and GluN2D allosteric potentiator CIQ on MK-801-induced deficit in PPI. WT, GluN2C HET and GluN2C KO mice were tested in PPI. MK-801 (0.15 mg/kg, ip) administration induced a deficit in PPI in WT, GluN2C HET and GluN2C KO mice. CIQ (20 mg/kg, ip) pre-administration prevented MK-801-induced deficit in PPI in GluN2C WT and HET but not in GluN2C KO mice. No effect of MK-801 was observed on startle amplitude in either genotype. Two-way repeated measures ANOVA revealed a significant decibel and drug treatment effect for all genotypes.: Post-hoc Dunnett’s test was conducted to compare Veh-Veh groups to other treatments at each decibel for each genotype. In WT and GluN2C HET at all decibel levels only Veh-MK-801 was significantly different from control Veh-Veh group. In case of GluN2C KO CIQ-MK-801 group was also found to be significantly lower than control Veh-Veh group at 74 and 84 dB suggesting that CIQ was not effective in preventing MK-801-induced impairment in PPI. *P < 0.05, **P < 0.01, ***P < 0.001.

NMDA channel blockers produce deficit in PPI which is relevant to sensorimotor gating deficit in schizophrenia. We have previously shown that a GluN2C/2D potentiator CIQ prevented MK-801-induced deficit in PPI in wildtype mice^29^, however the GluN2C or GluN2D subtype selectivity of this effect remains unknown. Since our results from PCP-induced hyperlocomotion which is a model of psychosis in schizophrenia demonstrated an important role of GluN2C, we tested whether prevention of MK-801-induced PPI deficit by CIQ is mediated via GluN2C subunit (Fig. 6). Two-way repeated ANOVA was conducted for each genotype with drug treatment as between-group factor and prepulse decibel intensity as within-group factor. A significant decibel effect was observed for all genotypes. Further a significant effect of treatment on PPI in wildtype [F(3,37) = 5.085, P = 0.0048], GluN2C HET [F(3,34) = 5.941, P = 0.002] and GluN2C KO [F(3,32) = 6.161, P = 0.002] was observed. Post-hoc Tukey’s test revealed significant impairment in the % PPI following MK-801 treatment at 2 or 3 decibel levels in wildtype [74 dB; P = 0.003, 78 dB; P = 0.0015, 84 dB; P = 0.047)], HET [74 dB; P = 0.058, 78 dB; P = 0.005 and 84 dB; P = 0.0004)] and KO [74 dB; P = 0.2216; 78 dB; P = 0.0463 and 84 dB; P = 0.046)]. Post-hoc Dunnett’s test was conducted to compare Veh-Veh groups to other treatments at each decibel. Within wildtype groups, only the Veh-MK-801 group was significantly lower than the control group (Veh-Veh) at 74 dB (P = 0.0015), 78 dB (P = 0.0008) and 84 dB (P = 0.0263), suggesting that CIQ (20 mg/kg, ip) effectively prevented the MK-801-induced impairment in PPI. A similar effect was observed within GluN2C HET groups at 74 dB (P = 0.0333), 78 dB (P = 0.0027) and 84 dB (P = 0.0002), suggesting that CIQ was effective in rescuing deficits in PPI similar to what we observed in the wildtype group. In case of the GluN2C KO group, there was no significant difference between Veh-MK-801 group compared to control group (Veh-Veh) at 74 dB but CIQ-MK-801 group was significantly lower than Veh-Veh group (P = 0.0369). At 78 dB Veh-MK-801 group was significantly lower than Veh-Veh group (P = 0.0262). Howeverat 84 dB, both vehicle-MK-801 (P = 0.0267) and CIQ-MK-801 (P = 0.0009) groups were found to have significantly lower PPI compared to Veh-Veh group. It is possible that the lower sample size in GluN2C KO may be responsible for difference in significance at each decibel level. Nonetheless, together the results in GluN2C KO suggest that CIQ was not effective in attenuating MK-801-induced PPI impairment at least at two decibel levels unlike that in WT and GluN2C HET. One-way ANOVA analysis of startle amplitude showed no significant effect of treatment on startle amplitude in either genotype.

## Discussion

In this study we compared the behaviors in mice with partial or complete ablation of GluN2C or GluN2D subunit. Unlike previous studies we used knockout mice generated using C57BL/6N strain ES cells by insertion of a cassette which renders loss of protein but doesn’t involve deletion of gene as used in previously mouse models. We found that several behaviors were similar to those reported earlier, however, there were also some differences. The most striking difference was in the responsiveness of NMDA channel blockers. Previous studies have demonstrated that GluN2D KO mice are completely unresponsive to NMDA channel blocker induced hyperlocomotion as well as other changes in neuronal function and oscillations^22–24^. In contrast, we found that GluN2D HET and KO were not resistant to NMDA channel blocker-induced hyperlocomotion.

### Effect of GluN2C or GluN2D ablation on mouse behavior

We and others have previously characterized behavioral phenotypes in the GluN2C KO mice^17–19^ (Supplementary Table 1). Several of the behaviors that we observed in the pure C57BL/6N strain of mice were similar to previous observations. However, we also observed certain differences possibly due to the innate differences in N and J strain of C57BL/6 mice as recently reported^35^ or due to difference in the method of generation of the knockout model which may affect potential intronic elements. In GluN2C HET and KO mice we observed no change in locomotor activity or time spent in the center as well as motor coordination in the rotarod test. These results are in accordance with previous findings^17,18^. We found a significant increase in startle amplitude in GluN2C HET but not GluN2C KO and no change in PPI in either genotype was observed. Interestingly, we have previously reported an increase in startle amplitude in GluN2C KO mice and a deficit in PPI in GluN2C HET^19^. A similar trend was also observed by Takeuchi *et al*.^33^ but this was not significant. Recent comparison between the C57BL/6N and C57BL/6J has found a significant difference in the startle amplitude and PPI among the two strains^35^ which may be responsible for the differences in the two studies. Nonetheless, our previous and current results suggest that partial ablation of GluN2C leads to greater behavioral change compared to complete ablation potentially because there may be more robust homoestatic compensatory changes in the KO model. We also found a significantly higher depression-like behavior in GluN2C KO which had higher immobile time in forced swim test. A similar trend was observed in our previous study but it did not reach significance^18^. Because the C57BL/6N strain exhibit higher anxiety-like behavior compared to C57BL/6J^35^ this may explain the detectable expression of depression-like behavior in the current study. We did not find a deficit in social interaction in the GluN2C KO mice similar to our previous finding^18^.

We found that GluN2D KO showed lower locomotor activity and lower number of entries into the central zone. No difference in motor coordination in rotarod was observed. Basal hypolocomotion in GluN2D KO is consistent with previous reports^20,21^ (Supplementary Table 2) which has been proposed to occur due to lower monoaminergic system function in the GluN2D KO mice^21^. Consistent with previous reports an increase in startle amplitude was observed in GluN2D HET and KO^24,33^, however, unlike previous reports we found that GluN2D HET and KO had lower PPI at some of the decibels tested. The higher depression-like behavior and normal social behavior in GluN2D KO mice were consistent with previous reports^21,36^. Thus, it appears that although many behaviors are replicated in GluN2D HET and KO in the current study there are also certain specific differences which may have substantial impact on our interpretation of role of this subunit in the CNS. These differences may arise due to subtle differences in mouse substrains or the gene construct in the knockout models. Another interesting observation is that gene-dosage does not directly correlate with certain behavioral deficit. For example, GluN2C HET exhibit increase in startle amplitude but not GluN2C KO. Similarly, a more robust effect on PPI is observed in GluN2D HET compared to GluN2D KO. As indicated above it is possible that complete deletion leads to stronger homeostatic changes thereby leads to normal behavior compared to heterozygous genotype.

### NMDA channel blocker effect and roles of GluN2C and GluN2D subunit

In the assessment of PCP-induced hyperlocomotion we found that GluN2C KO mice showed lower hyperlocomotion in response to PCP. Similarly, a significant reduction in PCP-induced hyperlocomotion was also observed in GluN2C KO on a strain with mixed background (Supplementary Fig. 1) which we have recently characterized^19^. Further, in contrast to GluN2C KO, the GluN2D KO mice were not resistant to PCP-induced hyperlocomotion and showed robust hyperlocomotion. This finding contrasts with previous studies which have found lack of NMDA channel blocker induced hyperlocomotion in GluN2D KO mice^22–24^. Although the precise reason for the difference in responsiveness to PCP in our and previous studies is unknown there were certain experimental variables to consider. We administered PCP intraperitoneally at a slightly higher dose than previous studies. We also found that GluN2D KO were not resistant to the hyperlocomotion induced by MK-801 thus the effect is not dependent on PCP. It was recently shown that ablation of GluN1 subunit from PV-neurons reduced NMDA channel blocker MK-801-induced locomotion but at the same time induced catatonic state which accounted for reduced locomotion^37^. Thus, there are limitations in the interpretation of locomotor activity alone in concluding the susceptibility to NMDA receptor antagonist. It is possible that GluN2D KO in previous reports did not exhibit hyperlocomotion in response to NMDA channel blockers but other stereotyped behaviors but this has not been reported. In the present study we were not able to address potential role of GluN2C and GluN2D deletion on the antidepressant efficacy of ketamine. We found minimal effect of ketamine on wildtype C57BL/6N mice strain on mobile events in the forced-swim test. Future studies using a broader range of conditions in which ketamine produces antidepressant effect are needed to address the role of individual subunits.

Finally, we identified that GluN2C subunit is important for the rescue of MK-801-induced deficit in PPI by CIQ. The GluN2C subunits are expressed in brain areas such as pontine reticular formation, mediodorsal thalamus, nucleus reticularis and in astrocytes in cortical regions^1,4,38^ which are relevant to schizophrenia circuitry and mediate startle and PPI^32,39,40^, suggesting that these receptors may at least partly regulate these processes. In our previous study, we found that MK-801 produced impairment in PPI in wildtype animals^29^. Similarly, in the present study intraperitoneal administration of MK-801 significantly decreased PPI in wildtype, GluN2C HET and GluN2C KO animals, this effect of MK-801 was prevented by CIQ in wildtype and GluN2C HET. However, in GluN2C KO animals CIQ-MK-801 group was significantly lower than Veh-Veh group at 74 and 84 dB. These results indicate that the facilitation of GluN2C-containing receptors may attenuate schizophrenia-like deficits induced by MK-801. It should be noted that despite a potential role of GluN2C-containing receptors in mediating effect of CIQ, we did not observe a deficit in PPI in GluN2C HET and KO. It is possible that homeostatic change during development in these genetic models may compensate for the loss of the receptor but acutely GluN2C-containing receptors may contribute significantly to PPI. Together, the findings from our studies demonstrate an important role of GluN2C subunit in schizophrenia-like psychotic and sensorimotor deficits.

## Materials and Methods

### Animal husbandry

We used the Grin2C^tm1(EGFP/cre/ERT2)Wtsi^ and Grin2D^tm1a(EUCOMM)Wtsi^ mouse line from Wellcome Trust Sanger Institute on pure C57BL/6N background. Behavioral procedures were performed on age matched 8–10 weeks old male wildtype, HET, and KO littermate mice. For some experiments, as noted, including the effect of CIQ on PPI, knockout mice in which a nβ-galactosidase reporter cassette replaced the GRIN2C gene as previously described were used^4,19^. Animals were group-housed on a 12:12 light/dark cycle with ad libitum access to food and water. At least 3 or more litters formed the subjects of each of the experimental group and experiments were conducted in at least 2 or more batches and assimilated. All procedures were approved by the Creighton University Institutional Animal Care and Use Committee and conformed to the NIH Guide for the Care and Use of Laboratory Animals.

### Tissue preparation and western blotting

Total protein or synaptoneurosome samples were prepared from cerebellar or thalamic tissue and western blotting was conducted as previously described^41,42^. Briefly, samples were resolved on SDS-PAGE gel and transferred onto nitrocellulose membrane. Membranes were blocked with 5% dry milk in Tris-buffered saline/Tween 20 (TBST) at room temperature for 1 hour and incubated with appropriate primary antibodies for overnight at 4 °C. The specific antibodies used were as follows; GluN2C (Neuromab, 75–411; RRID: AB_2531892 at 1:1000), GluN2D (Millipore, MAB5578, 1:2000), or β-actin (Santa Cruz Biotechnology, sc-69879, 1:4000). The blots were incubated in horse-radish peroxidase conjugated anti-mouse secondary antibody (Cell Signaling Technology, #7076, 1:2000) in 5% dry milk solution in TBST for 1 hour at room temperature followed by washing with TBST. Blots were developed using SuperSignal® West Pico chemiluminescent substrate (Thermo Scientific, Rockford, IL, USA) and processed using X-ray film processor model- BMI No 122106 (Brown’s Medical imaging, Omaha, NE, USA). The representative blots shown were not modified for exposure or contrast from the original X-ray films and were not assembled from cropped images. The images represent the entire blot of the indicated samples from ~250 to ~40 kDa.

### Drugs

MK-801 (Sigma-Aldrich), PCP (Sigma-Aldrich, St. Louis, MO, USA) and Ketamine HCl (Ketaset^®^, Zoetis Inc, Kalamazoo, MI) were dissolved in saline to the final concentrations and injected by ip route. CIQ (Tocris bioscience) was dissolved in dimethyl sulfoxide (DMSO) and injected by ip route.

### Behavioral analysis

All the animals were habituated to the experimental room and handled by the experimenter before the test day to reduce novelty induced anxiety. On the test day, animals were placed in the testing room 1 hr prior to the test. All the behavior testing was performed in between 10.00 am–14.00 pm. In between each animal, the apparatus was cleaned with 70% ethanol and air dried.

### Open field test

Open field test was performed in a custom-made square box (38 cm × 38 cm × 30 cm). Each animal was placed in the open field chamber and the behavior was videotaped for 15 min by using high resolution camera (Logitech) fitted to the ceiling of the experimental room. Total distance travelled in the box as well as the amount of time and distance traveled in the center area of the open field was scored by using AnyMaze video-tracking software (Stoelting, Wood Dale, IL, USA).

### Rotarod test

Rotarod test was performed by using the Rotamex apparatus (Columbus Instruments, Columbus, Ohio, USA.). The test was carried over five days, two trials per day with an inter-trial interval of 5 hr. On the first day, all animals were habituated to the rotarod for 5 min by placing the mice on the rod with no rotation. On second day, the mouse was placed on a rotating rod with a speed of 4 rotations per minute (rpm) for 5 min. On the third through fifth days, the animals were placed on accelerating rotarod with a starting speed of 4 rpm and reaching 44 rpm in 5 min. The latency (in seconds) to fall or turn completely with the rotating rod was noted by the digital meter of the apparatus.

### Locomotor activity

Locomotor activity was assessed in a custom-made circular open-field chamber (27.9 cm diameter × 35.6 cm wall height) bisected by two photobeams. Locomotion was counted via an automated photobeam break counter, indicating spatial movement when each photobeam was interrupted (Med Associates, Inc., St. Albans, VT, USA). The animals were placed in the chamber and the baseline activity was measured for 45 min. The animals were briefly removed from the chamber and were injected with saline or PCP (10 mg/kg, ip) and then placed back into the chamber. The locomotor behavior was recorded for 2 h. Total beam breaks after the administration of saline or PCP were measured.

### Prepulse inhibition

Startle activity was measured using an SR-LAB startle response system (San Diego Instruments, San Diego, CA, USA). The animal enclosure to measure acoustic startle response (ASR) was a transparent acrylic cylinder (size 12.7 cm × 3.81 cm for mice) fixed on a platform connected to a piezoelectric accelerometer that measures animal movements with an ultra-stable, hermetically sealed motion sensor using a 12-bit resolution. Above the cylinder was a speaker capable of producing noise up to 120 dB attached to programmable audio controls. The animal enclosure was situated in a sound attenuating isolation cabinet (38.1 cm × 35.56 cm × 45.72 cm) illuminated by a LED (San Diego Instruments). Mice were habituated in the startle chamber for two days. For PPI experiment, after 5 minutes of acclimation to the startle environment, the response to the startle stimulus alone (120 dB noise, 20 ms duration) and the effect of prepulse stimuli (74, 78 and 84 dB noise, 20 ms duration) delivered 100 ms before the onset of the startle stimulus (120 dB noise) were measured. The acoustic stimuli were superimposed on a 65 dB background noise. Each PPI session consisted of a total of 54 trials subdivided into 4 blocks. Blocks 1 and 4 were pulse alone trials (120 dB) consisting of four stimuli presentation. Blocks 2 and 3 consisted of prepulse and pulse alone trials. A total of 23 trials were presented during each of blocks 2 and 3 with five prepulse trials for each decibel and eight pulse alone trial. Trials within each block were presented in a pseudorandom order and were separated by an inter-trial interval ranging from 9 to 21 seconds. Measures of PPI were assessed referencing to the startle stimulus alone presentation as follows:

$$\begin{array}{c}{\rm{ \% }}\,{\rm{P}}{\rm{P}}{\rm{I}}=({\rm{m}}{\rm{e}}{\rm{a}}{\rm{n}}\,{\rm{s}}{\rm{t}}{\rm{a}}{\rm{r}}{\rm{t}}{\rm{l}}{\rm{e}}\,{\rm{r}}{\rm{e}}{\rm{s}}{\rm{p}}{\rm{o}}{\rm{n}}{\rm{s}}{\rm{e}}\,{\rm{t}}{\rm{o}}\,120\,{\rm{d}}{\rm{B}}\,{\rm{p}}{\rm{u}}{\rm{l}}{\rm{s}}{\rm{e}}\,{\rm{a}}{\rm{l}}{\rm{o}}{\rm{n}}{\rm{e}}-{\rm{m}}{\rm{e}}{\rm{a}}{\rm{n}}\,{\rm{s}}{\rm{t}}{\rm{a}}{\rm{r}}{\rm{t}}{\rm{l}}{\rm{e}}\,{\rm{r}}{\rm{e}}{\rm{s}}{\rm{p}}{\rm{o}}{\rm{n}}{\rm{s}}{\rm{e}}\\ \,\,{\rm{f}}{\rm{o}}{\rm{l}}{\rm{l}}{\rm{o}}{\rm{w}}{\rm{i}}{\rm{n}}{\rm{g}}\,{\rm{a}}\,{\rm{p}}{\rm{r}}{\rm{e}}{\rm{p}}{\rm{u}}{\rm{l}}{\rm{s}}{\rm{e}})/{\rm{m}}{\rm{e}}{\rm{a}}{\rm{n}}\,{\rm{s}}{\rm{t}}{\rm{a}}{\rm{r}}{\rm{t}}{\rm{l}}{\rm{e}}\,{\rm{r}}{\rm{e}}{\rm{s}}{\rm{p}}{\rm{o}}{\rm{n}}{\rm{s}}{\rm{e}}\,{\rm{t}}{\rm{o}}\,120\,{\rm{d}}{\rm{B}}\,{\rm{p}}{\rm{u}}{\rm{l}}{\rm{s}}{\rm{e}}\,{\rm{a}}{\rm{l}}{\rm{o}}{\rm{n}}{\rm{e}})\ast 100.\end{array}$$

For evaluation of effect of CIQ on MK-801-induced PPI deficit, CIQ (20 mg/kg, ip) was injected 30 minutes before beginning of PPI testing followed by MK-801 (0.15 mg/kg, ip) which was injected 15 minutes before beginning of PPI testing.

### Forced swim test

Forced swim test was conducted to assess depression-like behavior. The test was conducted in a glass cylinder measuring (30 cm height × 20 cm diameter). The cylinder was filled with the water (23–25 °C) to 15 cm of height. Each mouse was gently placed into the cylinder and swimming activity was videotaped for 6 min. The prerecorded videos were manually scored by the experimenter blind to the genotype and treatment. To avoid novelty-induced differences, first 2 min of the test were excluded and the videos were scored for last 4 min. The mobility was considered as any movement other than those necessary to maintain balance and to keep head above the water level. The data were represented as events mobile and immobile. To test the antidepressant effect of ketamine, mice were injected with ketamine (20 mg/kg, ip) 30 minutes prior to testing in forced swim test.

### Social interaction test

Social interaction test was performed as described previously^34^ with slight modifications. The sociability test apparatus comprised of a rectangular, three chambered plexiglass box. Each of the three chambers was 20 cm length × 40.5 cm width × 22 cm high. The dividing walls were made of clear plexiglass. The small doorways at center of each dividing wall allowed the access to each chamber. During the acclimatization, each chamber was isolated by using the dividing walls. The clear plexiglass cylindrical containers having holes were placed in center of right and left chamber. In one side, an unfamiliar (animate) mouse was enclosed in the clear plastic cylindrical container and the other side contains an empty container (referred as inanimate object). Subsequently, the experimental mouse was placed in the middle chamber and allowed to acclimatize for 5 min. Thereafter, the doors on either side were opened and the experimental mouse was allowed to explore animate and inanimate cylinders for 10 min. A circle 1 cm beyond the periphery of the plastic containers was marked. The time spent by the experimental mouse within this circle interacting with the inanimate- or animate-containing plastic container was recorded and reported.

### Statistical analysis

Data was analyzed by one-way analysis of variance (ANOVA) or repeated measures ANOVA followed by Dunnett’s or Bonferroni’s multiple comparison test. Data was expressed as mean ± standard error of means (SEM) and value of P < 0.05 was considered to be statistically significant in all the cases.

## Supplementary information

Supplementary information

## Data Availability

All relevant data are available from the authors.
