# Supplementary material for: Differential effect of NMDA receptor GluN2C and GluN2D subunit ablation on behavior and channel blocker-induced schizophrenia phenotypes
Source: Sci Rep. 2019 May 20;9:7572. doi: 10.1038/s41598-019-43957-2 (PMC6527682; doi:10.1038/s41598-019-43957-2)
Supplement: Supplementary file 1 — Supplementary information [file 41598_2019_43957_MOESM1_ESM.docx]

**Differential effect of NMDA receptor GluN2C and GluN2D subunit ablation on behavior and channel blocker-induced schizophrenia phenotypes**

Gajanan P. Shelkar^1#^, Ratnamala Pavuluri^1#^, Pauravi J. Gandhi^1#^, Aparna Ravikrishnan^1^, Dinesh Y. Gawande^1^, Jinxu Liu^1^, Dustin J. Stairs^2^, Rajesh R. Ugale^3^ and Shashank M. Dravid^1^


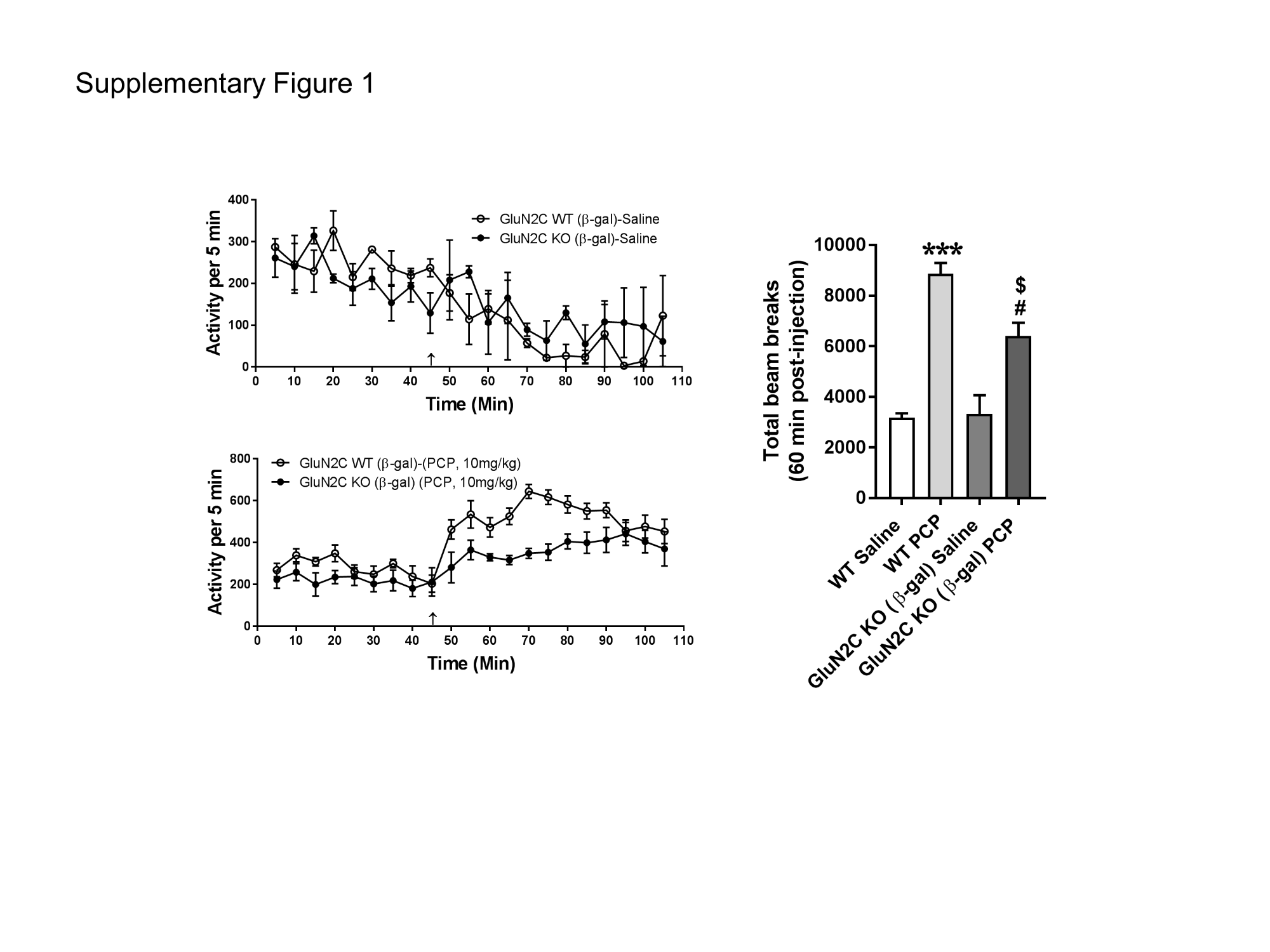


**Supplementary figure 1: Effect of PCP on locomotor activity in GluN2C KO (β-gal).** The WT and GluN2C KO (β-gal) animals were treated with saline or PCP and assessed for locomotor activity. Both WT and GluN2C KO (β-gal) mice showed significant increase in locomotor activity following PCP. ***P = 0.0002, ^#^P = 0.03 vs respective saline treatment, ^$^P = 0.01 vs WT PCP one-way ANOVA followed by Bonferonni post-hoc test).

| **Behavioral analysis of GluN2C knockout mouse line** | | | | | | | |
| --- | --- | --- | --- | --- | --- | --- | --- |
| **Paradigm** | **Parameter** | **Strain** | **Sex** | **Age** | **Drug and dose** | **Observations** | **Reference** |
| Open field test | Locomotor activity | C57B/6NTac | M | 6-8 Wks |  | No change in locomotor activity | Present study |
|  | Locomotor activity | C57B/6JJcl | Not specified | 5-6 Wks |  | No change in locomotor activity | ^[17]^Kadotani et al., 1996 |
|  | Locomotor activity | Mix C57B/6J (95%) + 129Sv/Ev | M | 4-8 Wks |  | No change in locomotor activity | ^[18]^Hillman et al., 2011 |
| Prepulse inhibition | Startle amplitude | C57B/6NTac | M | 6-8 Wks |  | Significant increase in startle amplitude in HET but no change in KO | Present study |
|  | Startle amplitude | C57B/6NJcl | M+F | 6-8 Wks |  | Trend for an increase in startle amplitude in HET | ^[33]^Takeuchi et al., 2001 |
|  | Startle amplitude | Mix C57B/6J (95%) + 129Sv/Ev | M | 6-8 Wks |  | Significant increase in startle amplitude in HET and KO | ^[19]^Gupta et al., 2016 |
|  | %PPI | C57B/6NTac | M | 6-8 Wks |  | No change | Present study |
|  | %PPI | Mix C57B/6J (95%) + 129Sv/Ev | M | 6-8 Wks |  | Significant reduction in PPI in HET at 82 dB | ^[19]^Gupta et al., 2016 |
|  | %PPI | C57B/6NJcl | M+F | 6-8 Wks |  | Trend for a reduction in PPI in HET at 78, 86 and 90 dB | ^[33]^Takeuchi et al., 2001 |
| Forced swim test | Immobility time | C57B/6NTac | M | 6-8 Wks |  | Significant increase in immobility in KO | Present study |
|  | Immobility time | Mix C57B/6J (95%) + 129Sv/Ev | M | 4-8 Wks |  | Trend for an increase in immobility in KO | ^[18]^Hillman et al., 2011 |
| Social interaction | Sociability | C57B/6NTac | M | 6-8 Wks |  | No change | Present study |
|  | Sociability | Mix C57B/6J (95%) + 129Sv/Ev | M | 4-8 Wks |  | No change | ^[18]^Hillman et al., 2011 |
| Drug induced hyper-  locomotion | Locomotion | C57B/6NTac | M | 6-8 Wks | PCP (10 mg/kg, ip) | Drug induced hyper-locomotion in HET,  less susceptible to PCP induced hyper-locomotion in KO | Present study |

**Supplementary table 1:** Summary of behavioral characterization of GluN2C knockout mice in present and previous studies.

| **Behavioral analysis of GluN2D knockout mouse line** | | | | | | | |
| --- | --- | --- | --- | --- | --- | --- | --- |
| **Paradigm** | **Parameter** | **Strain** | **Sex** | **Age** | **Drug and dose** | **Observations** | **Reference** |
| Open field test | Locomotor activity | C57B/6NTac | M | 6-8 Wks |  | Significant reduction in locomotor activity and center entries in KO | Present study |
|  | Locomotor activity | C57B/6NJcl | Not specified | P26- P28 |  | Significant reduction in locomotor activity in KO | ^[20]^Ikeda et al., 1995 |
|  | Locomotor activity | C57B/6NJcl | Not specified | 12 Wks |  | Significant reduction in locomotor activity in KO | ^[21]^Miyamoto et al., 2002 |
| Prepulse inhibition | Startle amplitude | C57B/6NTac | M | 6-8 Wks |  | Significant increase in startle amplitude in HET and KO | Present study |
|  | Startle amplitude | C57B/6NJcl | M+F | 6-8 Wks |  | Trend for an increase in startle amplitude in KO at 95-120 dB | ^[33]^Takeuchi et al., 2001 |
|  | PPI | C57B/6NTac | M | 6-8 Wks |  | Significant decrease in PPI in HET at 74, 78 and 84 and in KO at 78 | Present study |
|  | PPI | C57B/6NJcl | M+F | 6-8 Wks |  | Trend for an increase in PPI in HET at 78, 82, 86, and 90 dB and in KO at 90 dB | ^[33]^Takeuchi et al., 2001 |
| Forced swim test | Immobility time | C57B/6NTac | M | 6-8 Wks |  | Significant increase in immobility in KO | Present study |
|  | Immobility time | C57B/6NJcl | Not specified | 12 Wks |  | Significant decrease in immobility time in KO | ^[21]^Miyamoto et al., 2002 |
| Tail suspension test | Mobility time | C57B/6NJcl | M | 14 Wks |  | Significant reduction in mobility in KO | ^[36]^Yamamoto et al., 2017 |
| Sucrose preference test | Sucrose intake | C57B/6NJcl | M | 14 Wks |  | Significant reduction in sucrose intake in KO | ^[36]^Yamamoto et al., 2017 |
| Social interaction | Sociability | C57B/6NTac | M | 6-8 Wks |  | No change | Present study |
|  | Sociability | C57B/6NJcl | M | 14 Wks |  | No change | ^[36]^Yamamoto et al., 2017 |
| Drug-induced hyper-locomotion | Locomotion | C57B/6NTac | M | 6-8 Wks | PCP (10 mg/kg, ip) | Drug-induced hyperlocomotion similar to WT | Present study |
|  | Locomotion | C57B/6NJcl | M | 13-23 Wks | PCP (3 mg/kg, sc) | Lack of drug-induced hyperlocomotion | ^[22]^Hagino et al., 2010 |
|  | Locomotion | C57B/6NJcl | M | 14 Wks | Ketamine  (25 mg/kg, SC) | Lack of drug induced hyper-locomotion | ^[23]^Yamamoto et al., 2016 |
|  | Locomotion | C57B/6NJcl | M | 10-12 Wks | Ketamine  (30 mg/kg, SC) | Lack of drug induced hyper-locomotion | ^[24]^Sapkota et al., 2016 |
|  | Locomotion | C57B/6NTac | M | 6-8 Wks | MK801 (0.3 mg/kg, ip) | Drug induced hyper-locomotion similar to WT | Present study |

**Supplementary table 2:** Summary of behavioral characterization of GluN2D knockout mice in present and previous studies.
